# Mobile Device Accuracy for Step Counting Across Age Groups

**DOI:** 10.2196/mhealth.7870

**Published:** 2017-06-28

**Authors:** François Modave, Yi Guo, Jiang Bian, Matthew J Gurka, Alice Parish, Megan D Smith, Alexandra M Lee, Thomas W Buford

**Affiliations:** ^1^ University of Florida Department of Health Outcomes and Policy Gainesville, FL United States; ^2^ University of Florida Department of Aging and Geriatric Research Gainesville, FL United States

**Keywords:** mobile, devices, physical activity, weight reduction, adults

## Abstract

**Background:**

Only one in five American meets the physical activity recommendations of the Department of Health and Human Services. The proliferation of wearable devices and smartphones for physical activity tracking has led to an increasing number of interventions designed to facilitate regular physical activity, in particular to address the obesity epidemic, but also for cardiovascular disease patients, cancer survivors, and older adults. However, the inconsistent findings pertaining to the accuracy of wearable devices for step counting needs to be addressed, as well as factors known to affect gait (and thus potentially impact accuracy) such as age, body mass index (BMI), or leading arm.

**Objective:**

We aim to assess the accuracy of recent mobile devices for counting steps, across three different age groups.

**Methods:**

We recruited 60 participants in three age groups: 18-39 years, 40-64 years, and 65-84 years, who completed two separate 1000 step walks on a treadmill at a self-selected speed between 2 and 3 miles per hour. We tested two smartphones attached on each side of the waist, and five wrist-based devices worn on both wrists (2 devices on one wrist and 3 devices on the other), as well as the Actigraph wGT3X-BT, and swapped sides between each walk. All devices were swapped dominant-to-nondominant side and vice-versa between the two 1000 step walks. The number of steps was recorded with a tally counter. Age, sex, height, weight, and dominant hand were self-reported by each participant.

**Results:**

Among the 60 participants, 36 were female (60%) and 54 were right-handed (90%). Median age was 53 years (min=19, max=83), median BMI was 24.1 (min=18.4, max=39.6). There was no significant difference in left- and right-hand step counts by device. Our analyses show that the Fitbit Surge significantly undercounted steps across all age groups. Samsung Gear S2 significantly undercounted steps only for participants among the 40-64 year age group. Finally, the Nexus 6P significantly undercounted steps for the group ranging from 65-84 years.

**Conclusions:**

Our analysis shows that apart from the Fitbit Surge, most of the recent mobile devices we tested do not overcount or undercount steps in the 18-39-year-old age group, however some devices undercount steps in older age groups. This finding suggests that accuracy in step counting may be an issue with some popular wearable devices, and that age may be a factor in undercounting. These results are particularly important for clinical interventions using such devices and other activity trackers, in particular to balance energy requirements with energy expenditure in the context of a weight loss intervention program.

## Introduction

Obesity is a major health concern in the United States, with estimates of overweight or obese Americans >20 years old ranging between 68.5-75.3% [1,2]. Despite efforts to curb the obesity epidemic, its prevalence remains high [1-3]. Moreover, only 1 in 5 Americans meet the physical activity recommendations set forth by the Centers for Disease Control (CDC) of at least 150 minutes of moderate-intensity activity (defined as approximately 3-6 metabolic equivalents of task [METs]) or 75 minutes of vigorous-intensity exercise (>6 METs) per week [4]. The ubiquitous nature of mobile devices, including smartphones and wearable devices, makes them potentially useful to increase physical activity levels, and improve adherence to exercise programs. According to the Pew Research Center, approximately 80% of Americans own a smartphone [5]. Additionally, the landscape for wearable technology has changed drastically over the past 5 years, with nearly 400 devices commercially available [6] and over 127 million wearable devices sold in North America alone in 2016 [7]. Between 2013 and 2018, the wearable device market is expected to grow ten-fold, from under US $2 billion dollars to US $19 billion dollars [7]. The wide adoption of wearable technology in the United States offers unique ways to track behavior, and possibly to intervene effectively and efficiently to help users or patients adopt a healthy lifestyle.

There is mounting evidence that mobile health strategies and wearable devices could improve health behavior interventions, in particular for chronic conditions across the socioeconomic gradient [8-11] and across age groups [12]. Although it is not clear whether smartphone apps and wearable devices are effective for weight loss [13] or physical activity prescription [14,15], these devices may still be useful to increase physical activity participation levels, and thus could potentially improve quality of life regardless of weight loss outcomes [16-19]. Nonetheless, and despite technological advancements, it is still unclear how accurate recent smartphones and wearable devices are with respect to activity tracking, and what factors affect accuracy. For instance, Case et al [20] show that a convenient sample of wearable devices and smartphone apps are accurately counting steps, albeit in a young population sample (mean age 28.1 years, standard deviation [SD] 6.2). Wen et al [21] showed that step counting is also accurate in a small sample (5) but that activity duration, energy expenditure, and sleep patterns are not adequately captured by current devices. Recently Kroll et al [22] also showed that heart rate may not be accurately measured by wrist-worn devices, in particular if the user is not in sinus rhythm. The devices of interest do not measure step count directly, but do so by using tri-axial accelerometer data collected at the wrist or at the waist [23], and use proprietary algorithms to infer step count. Moreover, these algorithms assume a normal gait, which is not affected by pathologies or loss of lower limb strength. Therefore, where a device is worn (such as wrist or waist, dominant hand or nondominant hand) could affect step counts. Preliminary work suggests that devices measuring at the wrist tend to undercount steps in a laboratory setting compared to waist-based devices, but that in free-living conditions, the trend is reversed [24]. Additionally, at low speeds, accelerometers on commercial wearable devices may not be precise enough to accurately count steps [23], for instance for post-stroke patients who may need to wear wrist-based devices at the ankle [25], or for older users. Older patients exhibit loss of muscular strength, which can affect gait patterns [26-28], and consequently may lead to mobile devices either overcounting or undercounting steps in this specific population.

The discrepancies between such studies suggest that it is useful to assess what potential variables affect step count. It is not known whether user characteristics such as weight, height, gender, or age affect the accuracy of step counting for such tools. Age is a particularly interesting variable, given the evidence on gait changes among older adults [26,27], variations in accelerometry data in older adults [28], as well as the additional walking need of older adults [29-32]. Moreover, height, weight, and dominant hand are also variables of interest since the devices considered in this study do not count steps directly; rather, they infer step counts based on internal accelerometer data at the wrist or at the waist. Walking 10,000 steps per day is a widely recommended goal to meet the current guidelines of the CDC [33-35], even though it may fall short in terms of energy expenditure and health benefits [36]. Smartphones and wearable devices are both commonly used technologies to monitor and track physical activity, and could possibly help users adhere to a healthy lifestyle [37], so it is critical to properly assess the step counting accuracy of such commonly used devices, with a particular focus on age groups.

The purpose of this paper is to address this gap in the current literature for a representative set of five wrist-worn devices (Apple Watch, Samsung Gear S2, Garmin 735XT, Garmin Vivofit, Fitbit Surge), two smartphones (iPhone 6s Plus, Nexus 6P) and the research-grade ActiGraph wGT3X-BT. This selection was made to reflect the two most common mobile operating systems (OSs; namely Android and iOS), the range of price points, and the most commonly purchased device brands (Fitbit, Garmin) available on the market. To this effect, we model and assess the accuracy of recent smartphones and wearable devices across three age groups.

## Methods

### Device Selection

As of 2016, there are an estimated 394 wearable devices from 266 companies that are capable of activity tracking [6], not including smartphones or smartphone apps, with a majority of these devices being worn at the wrist. We selected a representative sample of the most recent wrist-based devices (2015 and later) and smartphones that counted steps without the need of an additional foot pod (small accelerometer device that can be affixed to shoe laces). Since foot pods measure walking or running cadence directly, they are typically more accurate than devices measuring at the wrist or at the hip. Most current devices can communicate with a foot pod using Bluetooth or Bluetooth Low Energy and thus can have greater accuracy. However, foot pods can be burdensome for the user. Therefore, we restricted this study to wrist-worn devices and smartphones (hip measurement). Additionally, we selected devices to reflect the most popular brands on the market (Garmin and Fitbit), as well as the various price points of such tools, ranging from under US $100 (Garmin Vivofit) to over US $400 (Garmin 735XT). Two mobile OSs currently share over 98% of the mobile OS market, with Android comprising over 80% of sales (multiple brands, multiple models) between 2009 and 2016, and Apple’s iPhone (multiple models) representing an additional 18% [38,39]. Consequently, we added both leading mobile OS’s newest devices (ie, the Android Huawei-manufactured Google Nexus 6P and the iPhone 6S Plus). Both devices include step counting capabilities. We also included the smart-watches of the leading mobile OSs (ie, the Apple Watch 2 for iOS, and the Samsung Gear S2 for Android). Finally, we included ActiGraph’s wGT3X-BT as a research-grade wearable device for physical activity. We decided to refrain from incorporating physical activity mobile apps in the study since the counts are intrinsically linked to each device’s internal step counter, and therefore it would be difficult to disentangle the measurements from the devices and measurements from the apps, especially given the proprietary black-box nature of such systems. Moreover, with over 165,000 apps for health and fitness alone [40], this approach fell beyond the scope of this study, but rather within the scope of an app evaluation [14,15].

### Participant Recruitment

After receiving approval from the University of Florida Institutional Review Board (IRB201601145), we recruited participants using flyers that were disseminated across campus. Twenty participants were recruited in each of the following age groups: 18-39 years, 40-64 years, and 65-84 years, for a total of 60 participants. Subjects were recruited among people without a contraindication to exercise, and who were able to walk comfortably on a treadmill for 20 minutes at a speed between 2 and 3 miles per hour.

### Research Procedures and Data Collection

The purpose and the protocol of the study were explained to participants, who were then consented by the study team (AL, MDS). Each participant received a US $10 gift card for participating in the study, and were instructed that they would be asked to do two walks of 1000 steps on a treadmill, at a self-selected speed between 2 and 3 miles per hour. Participants were instructed that the treadmill would be started at 2 miles per hour, upon which they would start walking without holding onto the treadmill, and steps would be recorded. The speed was progressively increased to an acceptable level by the study team (AL, MDS), as instructed by the participant. After consent, participants self-reported sex, age, height, weight, and dominant hand. In the first 1000-step walk, the Fitbit Surge, Garmin Vivofit, and Apple Watch were attached to the right wrist of the participants, and the Samsung Gear S2 and Garmin 735XT were attached to the left wrist. This choice was dictated by the width of each device. The iPhone 6S Plus was attached to the right hip with a belt clip, and the Nexus 6P was affixed to the left hip. Devices were then swapped right to left and vice versa in the second 1000-step walk. The Actigraph wGT3X-BT was kept centered at the back of the waist during both walks. The number of steps were tallied with a manual tally counter by one of the team members (AL, MDS). The number of steps for each device was recorded at the end of each walk. Additionally, the Apple Watch and the Samsung Gear S2 were not synchronized to their respective smartphones (iPhone 6S Plus and the Nexus 6P) to ensure reliability of the data.

### Statistical Methods

We computed summary statistics for the participants’ characteristics. To estimate the counted steps from each device while controlling for correlated observations and covariates, we fitted a repeated measures mixed-effects model, in which the participant was the independent sampling unit. The outcome of the model was steps counted by the devices (ie, the smartphones, the actigraph, or the wrist-based devices); the distribution of this outcome was not skewed. The predictor variables in the full model included age, sex, body mass index (BMI), dominant hand, device, age-by-variable interactions, and device-by-variable interactions. Age-by-variable interactions included age-by-sex, age-by-BMI, age-by-dominant hand, and age-by-device. Similarly, device-by-variable interactions included device-by-sex, device-by-BMI, and device-by-dominant hand. The order of the predictors was fixed in the order listed above. An unstructured covariance model was assumed, which accounted for unequal variance across devices. We used a backwards selection strategy [41] for the full model in every cell. Predictor variables were removed by considering added-last tests (based on Cronbach alpha=0.05) until we arrived at the reduced, final model. We then computed estimated steps and confidence intervals for each device from the final model.

In the model, age was categorized as: 18-39 years old, 40-64 years old, and 65-84 years old. Our preliminary analysis revealed that there was no significant difference in left- and right-hand step counts for each device. Therefore, we averaged the measurements obtained from the two walks for each participant-by-device for modeling. In addition, we set a cutoff of 250 steps as a likely point of device failure (less than 1 out of every 4 steps counted). All step outcomes less than 250 were excluded from the model. We chose to use BMI as a predictor in place of height and weight, as these two variables were highly correlated and would introduce collinearity to the model. We conducted all analyses using SAS 9.4 (SAS Institute, Cary, NC).

## Results

We summarized the characteristics of the study participants in Table 1. The age of our study participants ranged between 19 and 83 years, with an average of 49.5 (SD 19.4). Overall, 60% (36/60) of the study participants were female. This percentage is the highest in the 65-84 year old group, in which 73.7% (14/19) of the participants were female. Overall, 90% (54/60) of the study participants were right-handed. The BMI of the study participants ranged between 18.4 and 39.6, with an average of 25.2 (SD 4.6). The BMI was lowest in the 18-39 year old group with a mean of 23.0, and highest in the 65-84 year old group, with a mean of 27.0.

**Table 1:** Participant characteristics. One BMI observation was missing.

Characteristics		Total (N=60)	Age 18-39 (n=21)	Age 40-64 (n=20)	Age 65-84 (n=19)
Age, mean (SD)		49.5 (19.4)	26.2 (5.1)	53.7 (7.0)	70.9 (4.3)
**Sex, n (%)**					
	Female	36 (60.0)	11 (52.4)	11 (55.0)	14 (73.7)
	Male	24 (40.0)	10 (47.6)	9 (45.0)	5 (26.3)
**Dominant hand, n (%)**					
	Right	54 (90.0)	20 (95.2)	19 (95.0)	15 (79.0)
	Left	6 (10.0)	1 (4.8)	1 (5.0)	4 (21.0)
BMI, mean (SD)		25.2 (4.6)	23.0 (2.7)	25.7 (4.6)	27.0 (5.4)

**Table 2:** Steps by device by age group (averaged across two measurements).

Device	Age							
	18-39 (n=21)		40-64 (n=20)		65-84 (n=19)	
	Mean	SD	Mean	SD	Mean	SD
Actigraph	1003.9	6.3	986.1	44.4	995.0	43.4
Apple Watch	964.9	59.0	970.9	38.4	1015.9	118.8
Fitbit Surge	959.7	57.6	943.9	81.5	945.6	85.5
Garmin 735XT	987.8	37.9	994.0	29.4	978.7	38.2
Garmin Vivofit	994.5	11.5	992.3	22.6	953.5	170.3
iPhone 6S Plus	1021.2	186.4	1035.0	129.6	1018.0	56.9
Nexus 6P	997.1	41.3	988.3	26.1	900.9	158.3
Samsung Gear S2	988.0	16.0	959.4	84.2	969.1	47.9

**Table 3:** Final reduced model type 3 fixed effects.

Effect	Numerator Degrees of Freedom	Denominator Degrees of Freedom	F value	*P*-value
Age group	2	55.8	0.79	0.4599
Sex	1	48	0.05	0.8164
BMI	1	47	2.30	0.1358
Dominant hand	1	54.4	2.10	0.1532
Device	7	49.5	3.56	0.0036
Age group x device	14	76.3	2.00	0.0287

**Table 4:** Predicted means of steps for each device by age (adjusted for BMI, dominant hand, and sex).

Device	Age 18-39	Age 40-64	Age 65-84
Actigraph, mean (CI)	1008.7 (989.2, 1028.2)	997.2 (976.8, 1017.6)	1002.7(984.4, 1021.1)
Apple Watch, mean (CI)	970.2 (934.4, 1006.0)	980.1 (942.9, 1017.4)	1023.6 (987.0, 1060.2)
Fitbit Surge, mean (CI)	965.0 (930.0, 999.9)	950.8 (913.7, 988.0)	953.3 (917.5, 989.0)
Garmin 735XT, mean (CI)	993.9 (973.5, 1014.4)	1003.3 (983.5, 1023.2)	986.4 (967.7, 1005.0)
Garmin Vivofit, mean (CI)	999.8 (956.3, 1043.4)	1002.9 (957.4, 1048.3)	961.2 (916.3, 1006.2)
iPhone 6S Plus, mean (CI)	1026.5 (964.7, 1088.2)	1045.1 (980.4, 1109.8)	1025.7 (961.4, 1090.1)
Nexus 6P, mean (CI)	1002.4 (956.2, 1048.6)	981.8 (932.8, 1030.9)	908.6 (860.7, 956.4)
Samsung Gear S2, mean (CI)	993.3 (966.6, 1020.1)	966.7 (939.2, 994.3)	976.8 (950.1, 1003.6)

We summarized the step counting characteristics of the study devices in Table 2.

We summarized the results from the mixed-effects models in Table 3 and Table 4. In the final model, we identified one significant interaction after backwards selection: age-by-device (*P*=0.030; Table 3). The other interactions, including the device-by-BMI interaction, were not significant and therefore removed from the final model. Device was also a significant predictor of step count (*P*=0.004; Table 3).

Based on the final model, we produced model-based estimates of the steps counted by each device stratified by age group (Table 4). We considered undercounting as devices with counts that differed from 1000 in a statistically significantly fashion, with predicted means under the 1000 step target. Similarly, overcounting was considered a statistically significant count over 1000. The estimated steps from the Actigraph, Apple Watch, Garmin 735XT, Garmin Vivofit, and iPhone 6S Plus were not significantly different from 1000, across the age groups. Conversely, the Fitbit Surge consistently significantly undercounted steps. The estimated steps from Fitbit Surge for the 40-64 and 65-84 year old groups were 950.8 (95% CI 913.7-988.0) and 953.3 (95% CI 917.5-989.0) respectively, which were significantly lower than the targeted 1000 steps.

The steps counted by the Fitbit Surge for the 18-39 age group were 965.0 (95% CI 930.0-999.9), which is much closer, but still significantly lower than 1000. In addition, the Nexus 6P undercounted steps in the 65-84 year old group, with an estimated count of 908.6 steps (95% CI 860.7, 956.4). The Samsung Gear S2 undercounted steps in the 40-64 year old group, with an estimated count of 966.7 steps (95% CI 939.2, 994.3). However, the same device did not significantly undercount steps for the older age group, with an estimated count of 976.0 steps (95% CI 950.1, 1003.6).

## Discussion

### Principal Findings

The ubiquity of smartphones and other wearable devices, and their various physical activity tracking functionalities, have led to an increasing reliance on such devices as tools for participation in exercise programs. Such functionalities include step tracking, global positioning system functions (eg, distance, pace, elevation, map), heart-rate monitoring (either wrist-based, or with a chest strap), or calorie expenditure. Although some evidence suggests that step-counting is accurate for some wrist-worn devices and smartphone apps [20,21], this is not consistent across all walking speeds, in particular lower speeds [23], or whether devices are worn at the wrist or waist [24]. Given the proprietary nature of algorithms inferring step counts from tri-axial accelerometer data, it was important to identify variables that potential impact the step count accuracy of such devices, in particular age, height, weight, and dominant or nondominant hand.

Our study indicates that height, weight, BMI, and dominant hand do not seem to impact the accuracy of step-counting devices. Conversely, our results suggest that the Fitbit Surge undercounted steps for all age groups, the Nexus 6P underestimated step counts for the 65-84 year old group, and the Samsung Gear S2 underestimated step counts for the 40-64 year old age group, but not the older age group (Table 4). Our hypothesis is that subtle gait changes and slower walking among older populations could explain why some devices tend to undercount steps in these groups. This theory is consistent with the findings of Fortune et al [23] linking walking speed and accuracy. Therefore, device manufacturers should ensure that algorithms inferring step counts from tri-axial accelerometer data be updated to account for such subtle changes. However, the Samsung Gear S2 only underreported step counts in the middle age group. This result is somewhat surprising, as we would anticipate that the devices underestimating step counts would perform worse in the older age group than in the middle group, if the main factor affecting count was gait changes associated to aging. A possible explanation is that the level of conditioning could be a confounding factor in our study, as strength and endurance training affect gait in older age groups [31]. Indeed, lack of strength in older adults is associated with gait changes [26-28]; this explanation also remains consistent with the findings in Fortune et al [23]. Therefore, additional work is needed when controlling for physical fitness levels. Additionally, unlike Tudor-Locke et al [24], we did not observe significant differences in step counting between waist- and wrist-based devices. Although Case et al [20] report good accuracy for their devices, their population sample was significantly younger, and their convenient device selection did not intersect with ours. Moreover, in previous studies [20,23,24] all devices used were at least 2 years old; the difference observed could be explained by technological and/or algorithmic changes in the devices used. Finally, Wen et al [21] reported that step counting for their choice of devices is accurate. However, the sample size of participants in that study was significantly smaller, and their study focused on longitudinal consistency (eg, internal validity of devices) rather than comparison between devices.

A major strength of this study is that, to the best of our knowledge, it is the first that evaluates the impact of age, BMI, and dominant hand on the accuracy of the newer generation of wearable devices and smartphones with respect to step counting. Although BMI and dominant hand do not appear to impact the ability of devices to estimate step counts, age does affect estimates of step counts for some devices. Therefore, additional work needs to be done to evaluate the impact of wrist patterns and gait on the accuracy of step counting, and explore what other potential factors influence the results. Nonetheless, from a physical activity program adherence and weight loss perspective, one could argue that since less accurate devices tend to underestimate step counts, they should still be recommended for tracking steps, and could lead to additional exercise.

### Limitations

A potential weakness of the study is that we tested step counting in idealized conditions, indoor, on a treadmill. In real-world conditions, especially difficult terrain, we may see far more variation in step counts, given the changes in gait and wrist movements. Additionally, it is not uncommon to see different gaits between normal walking conditions versus walking on a treadmill.

### Conclusion

Over the past 5 years, wearable devices, smartphones, and apps have become more ubiquitous, and have become widely recommended tools of behavioral change for weight loss by the general press, the health and fitness industry, and health care providers. In this study, we evaluated the accuracy of a selection of recently available wearable wrist-worn devices and smartphones with respect to step counting, as well as the impact of several variables of interest, most notably age. Our final reduced model after backward selection shows that BMI, height, weight, and dominant hand do not seem to impact the accuracy of step count. However, age does affect accuracy, and some devices tend to underestimate the number of steps walked by older users of wearable devices. This finding may be a minor issue for people trying to lose weight by adhering to a 10,000-step walking program, as they may walk more than planned. However, older and/or slower participants focusing on increasing physical activity may be negatively affected, and may struggle mentally if they fall short of 10,000 steps. What is not clear yet is whether current levels of physical fitness and activity impact the accuracy of such devices; this warrants further investigation.

## Abbreviations

BMI: body mass index
CDC: Centers for Disease Control
MET: metabolic equivalent of task
OS: operating system
SD: standard deviation
